# A Versatile and Rapidly Deployable Device to Enable Spatiotemporal Observations of the Sessile Microbes and Environmental Surfaces

**DOI:** 10.1264/jsme2.ME16161

**Published:** 2017-03-17

**Authors:** Tatsunori Kiyokawa, Ryo Usuba, Nozomu Obana, Masatoshi Yokokawa, Masanori Toyofuku, Hiroaki Suzuki, Nobuhiko Nomura

**Affiliations:** 1 Graduate School of Life and Environmental Sciences, University of Tsukuba1–1–1 Tennodai, Tsukuba, Ibaraki 305–8572Japan; 2 Graduate School of Pure and Applied Sciences, University of Tsukuba1–1–1 Tennodai, Tsukuba, Ibaraki 305–8573Japan; 3 Faculty of Life and Environmental Sciences, University of Tsukuba1–1–1 Tennodai, Tsukuba, Ibaraki 305–8572Japan; 4 Faculty of Pure and Applied Sciences, University of Tsukuba1–1–1 Tennodai, Tsukuba, Ibaraki 305–8573Japan; 5 Department of Plant and Microbial Biology, University of ZurichZürich 8008Switzerland

**Keywords:** flow channel device, real-time imaging, three-dimensional imaging, biofilms, microbe-surface interactions

## Abstract

Although microbes typically associate with surfaces, detailed observations of surface-associated microbes on natural substrata are technically challenging. We herein introduce a flow channel device named the Stickable Flow Device, which is easily configurable and deployable on various surfaces for the microscopic imaging of environmental microbes. We demonstrated the utility of this device by creating a flow channel on different types of surfaces including live leaves. This device enables the real-time imaging of bacterial biofilms and their substrata. The Stickable Flow Device expands the limits of conventional real-time imaging systems, thereby contributing to a deeper understanding of microbe-surface interactions on various surfaces.

Most known microbes found in the environment associate with surfaces. These surface-associated bacteria form multicellular communities called biofilms (4, 7). The surfaces themselves are as diverse as the environment may produce, including biotic and abiotic materials. Our understanding of biofilm lifestyles has markedly increased over the past few decades; however, many aspects of bacterial interactions with natural substrata remain unknown or poorly understood. One reason for this may be attributed to the limitations of conventional real-time imaging systems capable of observing sessile microbes on environmental surfaces; observations are limited to cover glasses. For example, confocal laser scanning microscopy (CLSM) has been extensively used to image biofilm growth within flow channels with glass bottoms that allow the continuous flow of growth medium; these channels are called flow cells (1, 8, 14). They are particularly suited to the real-time imaging of surface-attached microbes because continuous flow carries away planktonic cells. Despite its utility, this technique has a major limitation in that the microbes being observed must be attached to a surface, which is typically glass. However, in the actual environment, most bacteria adhere to and form biofilms on various surfaces other than glass. Substratum characteristics such as charge and hydrophobicity significantly influence microbial attachment to surfaces (5). Thus, systems that enable continuous flow while simultaneously accommodating different substrata have the potential to facilitate advances in microbial ecology in more natural environments.

We herein present an easy-to-configure flow channel device (named the Stickable Flow Device) that is deployable on a number of disparate surfaces in order to enable the live imaging of environmental microbes (Fig. S1). We demonstrate the utility of this device by creating a flow channel on stainless steel, plastic, wood, raw meat, and the surface of a live leaf. The Stickable Flow Device enables the non-invasive *in situ* three-dimensional (3D) confocal and real-time imaging of sessile microbes and their substrata.

We fabricated the flow device by cutting channels into water-resistant sponge double-sided tape with an acrylic adhesive (NICETACK NW-G15S; NICHIBAN, Tokyo, Japan). The channel length, width, and height were 20 mm, 1 mm, and 1 mm, respectively. We used a Rayjet laser cutter (Trotec Laser, Canton, MI, USA) to precisely generate channels in the double-sided tape. A schematic of the device attached to a leaf is shown in Fig. 1A and the flow pathway is shown in Fig. 1B. Since the channel walls are made from flexible sponge tape, the device was easily mounted on different materials with greatly varying surface chemistries and Young’s moduli including biotic substrata such as the surfaces of leaves, wood, and meat (Fig. 1C, D, E, F, and G). During experiments conducted in this study, water leakage was not observed in any sample. Channel reconfiguration according to usage was also easy using the laser cutter; images of multiple flow-channels, crossed flow-channels, and winding flow-channels are shown in Fig. S2.

In order to ensure the comparability of data obtained with the Stickable Flow Device, we tracked the biofilm formation of *Pseudomonas aeruginosa* tagged with Enhanced Green Fluorescent Protein (EGFP) in our flow device adhered to glass as a control. We used *P. aeruginosa* as a model biofilm-forming Gram-negative bacterium (Table S1). *P. aeruginosa* is known to adhere to and form biofilms on various surfaces (15). After inoculating *P. aeruginosa* into flowing medium, it irreversibly attached to the glass surface, formed microcolonies, and eventually generated large mushroom-like shape biofilm structures (Fig. S3); this is the typical biofilm lifecycle reported for *P. aeruginosa* (16). After confirming that these biofilms grew in the control experiment, we then deployed the Stickable Flow Device on various surfaces including stainless steel (Stainless steel plate SUS430; ASONE, Osaka, Japan), plastic (Polyethylene plate; ASONE), wood (Wooden name plates; DAISO, Hiroshima, Japan), and raw meat (purchased from a local grocery store) to demonstrate the utility of this device. In order to simultaneously visualize surface-associated microbes and their substrata, we imaged *P. aeruginosa* and the attached surfaces with CLSM and continuous-optimizing confocal reflection microscopy (COCRM), respectively (19). COCRM is a technique that takes advantage of reflected light to non-destructively take 3D images without staining (9, 10, 16, 19, 20). It is particularly useful for imaging the surfaces to which bacteria adhere. In our flow channels, we used CLSM to image EGFP-tagged *P. aeruginosa* in combination with COCRM to visualize surface structures. We found that biofilm structures were very similar on abiotic surfaces such as metal and plastic: bacteria and mushroom-like structures covered most of the field of view (Fig. 2A and B). In contrast, on biotic surfaces such as wood and meat, biofilm structures significantly differed. On wood, large heterogeneous biofilms developed interspersed on the protruding fibrous surface (Fig. 2C), while the entire surface on raw meat was covered by a thin biofilm (Fig. 2D). In order to examine differences in the biofilm formation process, attachment and microcolony formation processes were observed (Fig. S4). These processes on stainless steel and plastic surfaces were very similar (Fig. S4A, B, C, and D). On the wood surface, we found that bacteria initially attached uniformly (Fig. S4E and S5), then microcolonies formed specifically on the protruding fibrous surface (Fig. S4F). On the other hand, we observed a smaller number of bacterial cells attached to the meat surface (Fig. S4G and H). Thus, differences in biofilm structures resulted from differences in the attachment or microcolony formation process. These processes are known to be significantly influenced by surface roughness, surface charge, and hydrophobicity, which affects fluid flow velocity and bacterial surface motility (6, 7, 13, 15). Although we used small quantities of samples in our tests, this system may be used to compare microbial behavior on different surfaces under variable flow conditions and times. Using COCRM, we were able to visualize the 3D erosion of a surface material (9). Thus, the combination of COCRM with the Stickable Flow Device may enable the simultaneous imaging of microbial behavior, revealing dynamic changes in surface architecture, such as metal corrosion, under controlled flow conditions.

In order to demonstrate that the Stickable Flow Device is capable of the real-time imaging of biofilms and substrata, we tracked morphological changes in a biofilm that adhered to a stainless steel surface under controlled flow conditions. We observed a monotonic decrease in the biofilm biomass at a fixed spatial point under higher flow conditions in the presence of the non-ionic detergent, Tween 20 (Fig. 2E and S6). This result is an example of the utility that our device possesses and shows that it will enable a better understanding of how environmental conditions alter biofilm formation.

In order to simulate the deployment of the Stickable Flow Device in the environment, we applied it to the surface of a leaf of a living plant (*Setaria viridis*; collected from vacant land at the University of Tsukuba, Ibaraki, Japan) in order to observe bacterial growth. A number of plant pathogens attach to leaf surfaces and form biofilms, through which they invade plant apoplasts through open stomata (12). Confocal images of biofilms that adhered to the leaf surface are shown in Fig. 2F. Notably, an increase was observed in biofilm thickness immediately above the stomata (see arrows in Fig. 2F). A cross-sectional view of the leaf revealed the actual invasion of bacterial cells into apoplastic spaces, located under the stomata (Fig. 2G and S7). These results demonstrate the power of our system and its versatility as a 3D spatial imaging tool to enable heretofore technically challenging measurements that will shed light on plant-bacteria interactions. Furthermore, the Stickable Flow Device may be applied to other parts of the plant, such as the roots. Microbial communities established on plant roots play important roles in promoting plant growth and suppressing soil-borne diseases (2, 17). A deeper understanding of the plant-bacteria microbiome will have a direct impact in the field of agriculture and may provide a novel insight into how to regulate plant microbiomes in cash crops. This understanding begins with non-invasive visualization. More broadly, the visualization of sessile microbes on the skin of animals and on food may result in the mechanisms underlying bacterial infection as well as the ecological role of epibiotic microbes being clarified (3, 11, 18).

In conclusion, we herein demonstrate that the Stickable Flow Device developed in this study enables the rapid and robust deployment of a flow channel on surfaces as different as animal flesh, metal, and plastic. Combining our device with high spatial and temporal resolution imaging such as COCRM and CLSM will enable the non-invasive visualization of microbes under actual environmental settings. In the present study, we used *P. aeruginosa* as a model microbe; however, this system may be used to visualize other microbes. As our understanding of microbial ecology expands, it is becoming increasingly clear that the environment greatly affects microbial lifestyles. In order to gain a more complete understanding of microorganisms, we need to begin investigating their behavior in their natural context, which will require the development of environmentally deployable systems.

## Supplementary Information











## Figures and Tables

**Fig. 1:**
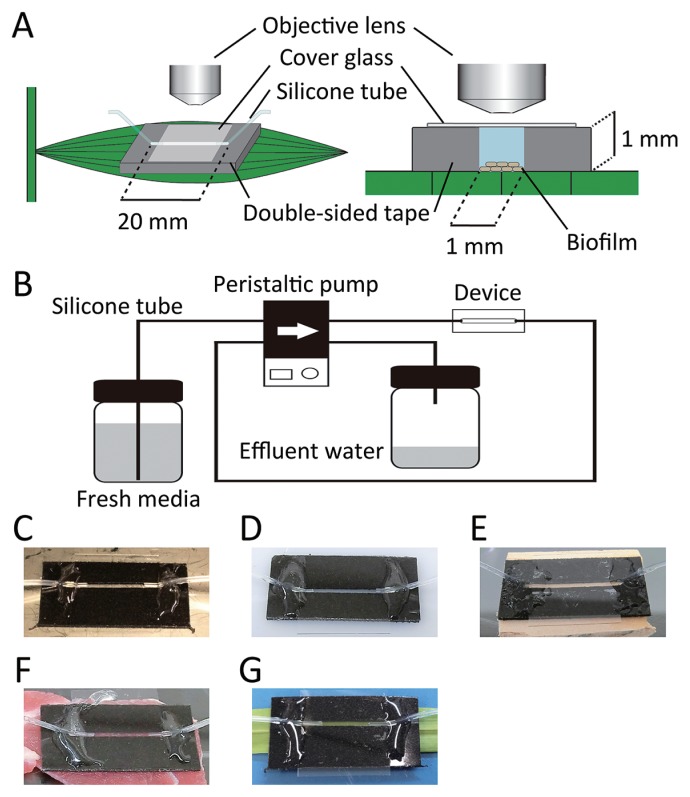
(A) Schematic overview (left) and cross-sectional view (right) of the Stickable Flow Device used in this study. Surface-attached microbes were observed through a cover-glass window using a microscope. (B) Flow pathway through the device. Fresh medium was continuously supplied to the device through a silicone tube and effluent water was drained through the outlet tube with a peristaltic pump (Ismatec, Glattbrugg, Switzerland). Images of the Stickable Flow Device attached to (C) stainless steel, (D) plastic, (E) wood, (F) raw meat, and a (G) leaf.

**Fig. 2:**
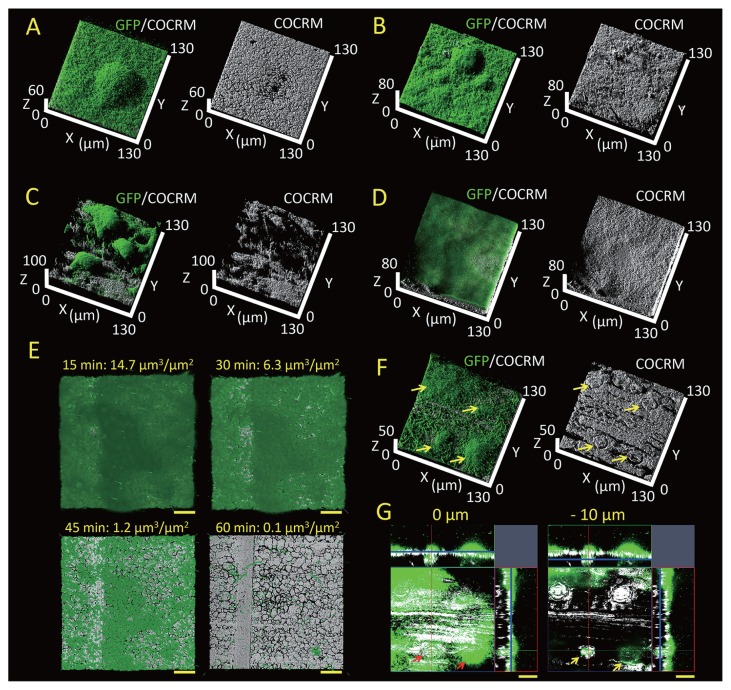
Observation of *P. aeruginosa* tagged with EGFP grown in the Stickable Flow Device. *P. aeruginosa* biofilm structures on (A) stainless steel, (B) plastic, (C) wood, and (D) raw meat surfaces. *P. aeruginosa* tagged with EGFP was grown under flow conditions in 1/5 MHB medium (Dilute the MHB medium (Becton, Dickinson, Sparks, MD, USA) 5 times with distilled water) supplemented with 20 mM KNO_3_ (Wako Pure Chemical Industries, Osaka, Japan) and 100 μg mL^−1^ tetracycline (Sigma Aldrich, St. Louis, MO, USA) at 25°C for 3 d. The mean flow velocity in the device was 0.5 mm s^−1^. The upright confocal laser scanning microscope LSM5 PASCAL (Carl Zeiss, Oberkochen, Germany) incorporated with a 63×/0.9 numerical aperture W N-Achroplan water dipping objective lens (Carl Zeiss) was used to acquire confocal microscopic images. EGFP (Green) were excited by an argon laser (488 nm) and detected with a 505-to-530 nm band-pass filter. Material surfaces (white) were illuminated with an argon laser (514 nm), and reflected light was collected through a 505-to-530 nm band-pass filter. (E) Real-time imaging of 2-d-old biofilm detachment of *P. aeruginosa*. Biofilms formed on stainless steel were exposed to a higher flow velocity (1.5 mm s^−1^) in the presence of 0.1% (v/v) Tween 20, and tracked over time. The elapsed time after the treatment is shown (top left). Image analysis for biomass quantification (top right), CLSM images of *P. aeruginosa* tagged with EGFP were assessed using volumetric analyses with IMARIS version 7.2.1 software (Bitplane, Zurich, Switzerland). The scale bar indicates 20 μm. See supporting movie Fig. S6 for the image sequence indicated. (F) *P. aeruginosa* formed biofilms on top of the stomata. *P. aeruginosa* tagged with EGFP was grown under flow conditions in 1/5 MHB medium supplemented with 20 mM KNO_3_ and images of biofilms and leaf surface structures. Horizontally sectioned images (square) and vertically sectioned (rectangle) images were shown. The 100 μg mL^−1^ tetracycline at 25°C for 3 d. This experiment was performed under dark conditions. Yellow arrows indicate stomata. (G) Cross-sectional blue line in the rectangular images marks the location of the horizontal section. Red arrows indicate stomata. Yellow arrows indicate the invasion of bacterial cells (green) to the apoplastic space located under the stomata. The Z-position from the outer leaf surface (top). The scale bar indicates 20 μm.
