# Supplementary material for: A Versatile and Rapidly Deployable Device to Enable Spatiotemporal Observations of the Sessile Microbes and Environmental Surfaces
Source: Microbes Environ. 2017 Mar 17;32(1):88–91. doi: 10.1264/jsme2.ME16161 (PMC5371081; doi:10.1264/jsme2.ME16161)
Supplement: Supplementary file 1 [file 32_88_s3.pdf]

Constructions of plasmid and strain

The bacterial strains and plasmids used in this study are listed in Table S1. *P. aeruginosa* and *Escherichia coli* were cultured at 37°C in LB Lennox medium (Nacalai, Kyoto, Japan) with shaking at 200 rpm or LB plates containing 1.5% (w/v) agar. We used tetracycline at a concentration of 100 μg ml^{-1} for culturing *P. aeruginosa*, while for *E. coli*, we used a concentration of 10 μg ml^{-1}.

The *P. aeruginosa* PAO1 tagged with EGFP strain constitutively expresses EGFP. We constructed it as follows: 1) pEGFP (Clontech, Palo Alto, CA, USA) was digested with BamHI and EcoRI to purify the *egfp* fragment; 2) the *egfp* fragment was cloned into a BamHI/EcoRI-treated pUCP27 plasmid (2); 3) this plasmid (pUCP27 EGFP) was introduced into *P. aeruginosa* PAO1 through electroporation.

Table S1. Bacterial strains and plasmids

Strain or plasmid	Details	Reference
Strains		
P. aeruginosa PAO1	Wild-type strain	1
E. coli DH5alpha	*E. coli* cloning strain	TaKaRa
Plasmids		
pEGFP	Plasmid harbouring *egfp*	Clontech
pUCP27	*E. coli* and *P. aeruginosa* shuttle plasmid for gene explession, TET^r	2
pUCP27 EGFP	pUCP27 derived EGFP expression vector	This study

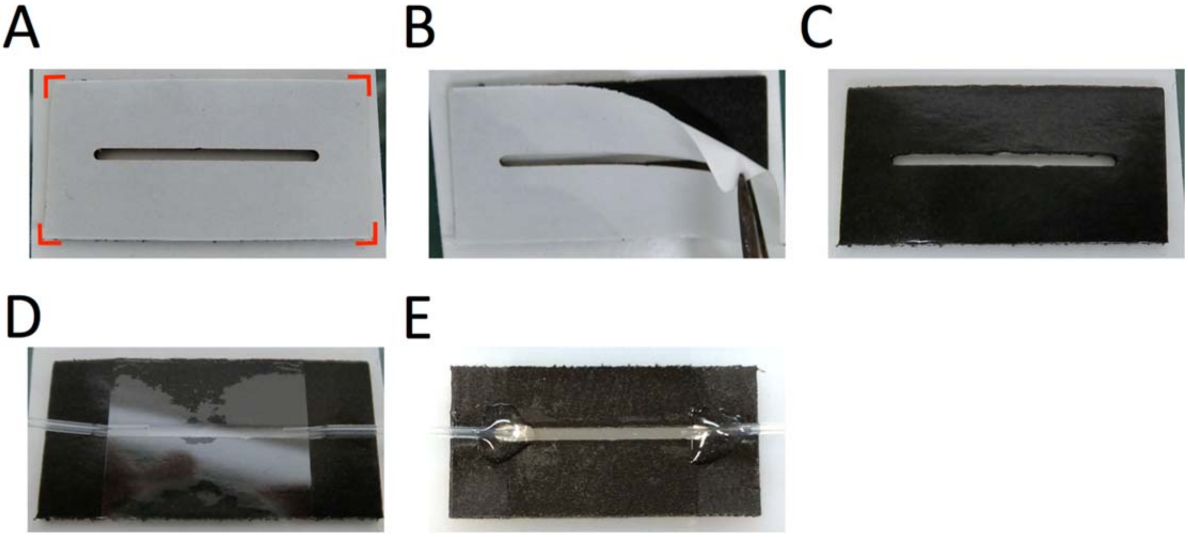

Fig. S1. Process sequence for the fabrication of the Stickable Flow Device. (A) The double-sided tape with flow channel. Red edge frames indicate outline of the double-sided tape. (B and C) Peeling off a single side of release paper from the tape. (D) Sticking a cover glass and silicone tubes on the tape. The edges of the silicone tubes are inserted into the flow channel from gaps between the double-sided tape and the cover glass. The tubes are fixed with an adhesive not to come off. (E) Peeling off a rest release paper and sticking the Stickable Flow Device on plastic plate in the picture. The channel length and width were respectively 20 mm and 1 mm.

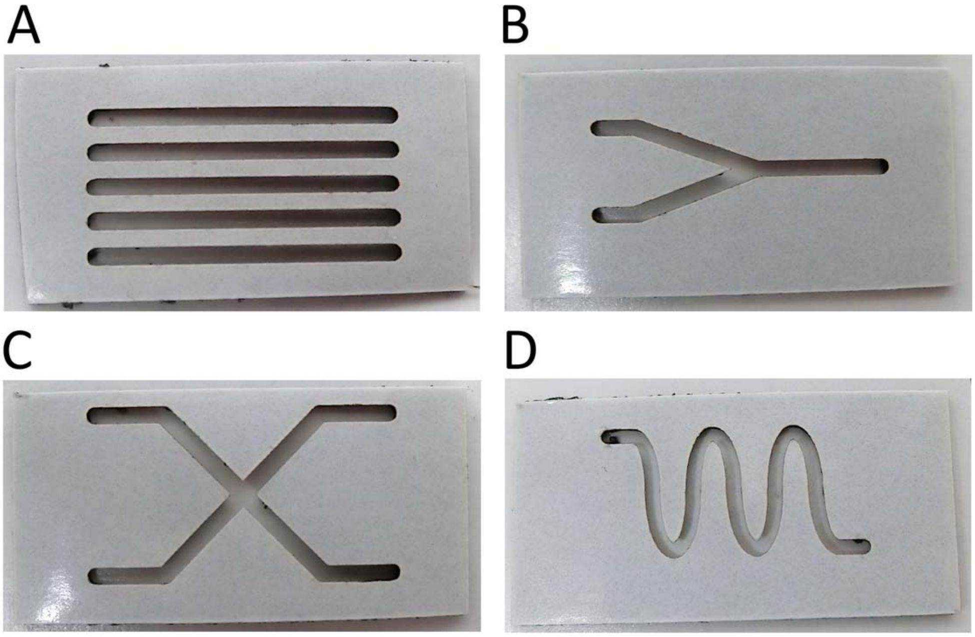

Fig. S2. Various flow channels constructed by using laser cutter. (A) Multiple flow channels, (B) Y-shape flow channel, (C) crossed flow channels, and (D) winding flow channel were shown. The width of each flow channels was 1 mm.

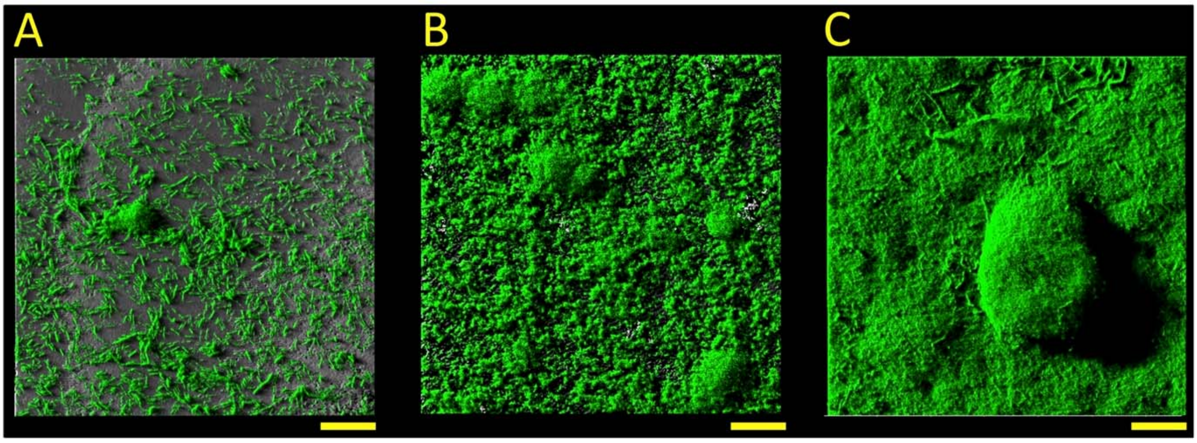

Fig. S3. Observation of biofilm developmental process (attachment, microcolony formation, and typical mushroom-like shape biofilm formation) on the glass surface (MATSUNAMI GLASS, Osaka, Japan) by using the Stickable Flow Device. *P. aeruginosa* tagged with EGFP were grown under flow conditions in 1/5MHB medium supplemented with 20 mM KNO_3 and 100 μg ml^{-1} tetracycline for (A) 1days, (B) 2days, (C) 3days. Mean flow velocity in the device was 0.5 mm s^{-1}. EGFP (Green) were excited by an argon laser (488 nm) and detected with a 505-to-530 nm band-pass filter. Glass surfaces (white) were illuminated with an argon laser (514 nm), and reflected light was collected through a 505-to-530 nm band-pass filter. Scale bar indicates 20 μm.

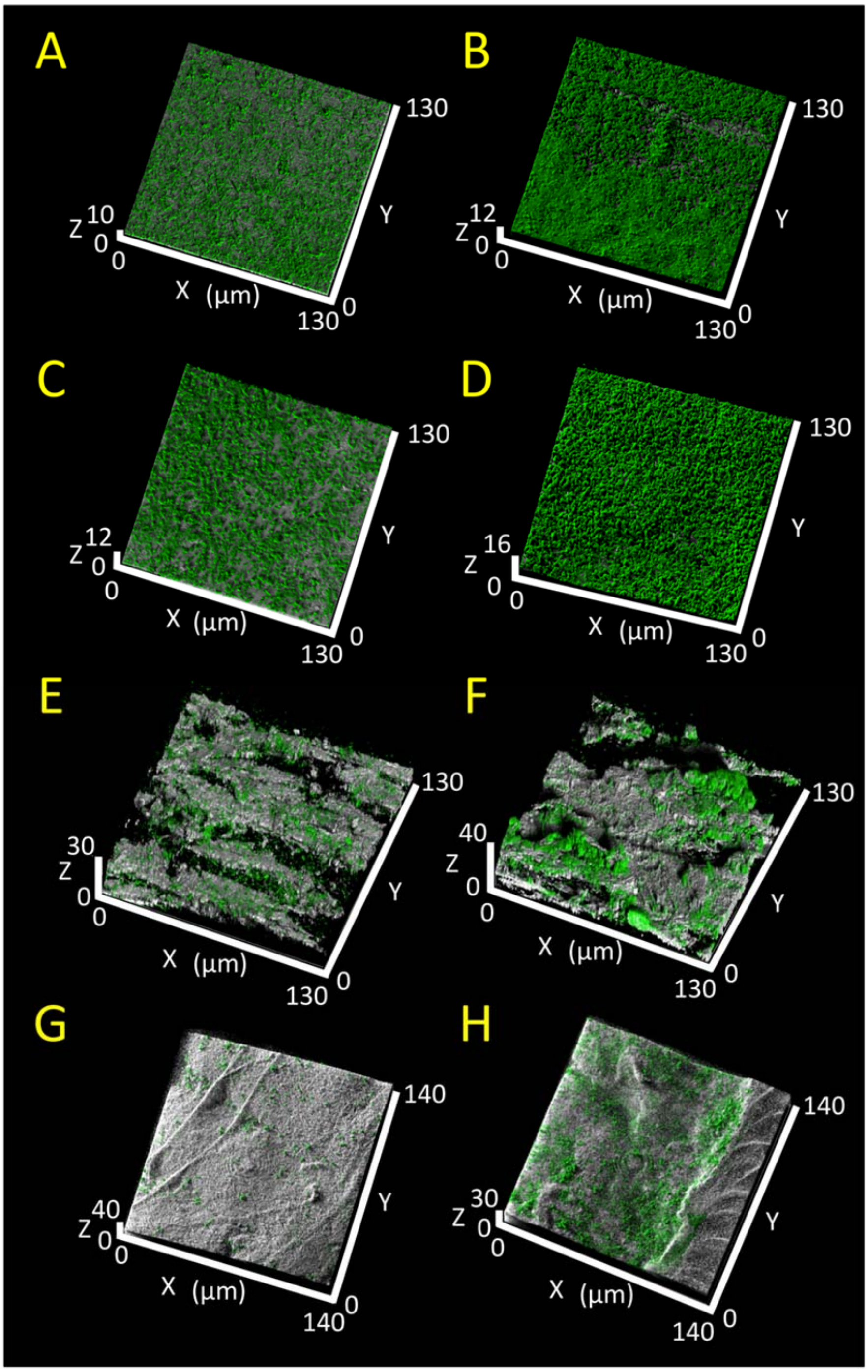

Fig. S4. Observation of biofilm developmental process in the Stickable Flow Device. *P. aeruginosa* were grown for 1 day (A, C, E, and G) and 2 days (B, D, F, and H) on stainless steel (A and B), plastic (C and D), wood (E and F), and raw meat (G and H) surfaces. Green color indicated *P. aeruginosa* tagged with EGFP. White color indicated substratum surfaces.

See “FigS5.mov” (for Mac users) or “FigS5.avi” (for windows users).

Fig. S5. 3D view of Fig. S4E. *P. aeruginosa* uniformly attached to the wood surface. Blue grid lines are drawn at 20 μm intervals. Green color indicated *P. aeruginosa* tagged with EGFP. White color indicated substratum surfaces.

See “FigS6.mov” (for Mac users) or “FigS6.avi” (for windows users).

Fig. S6. Real-time imaging of biofilm detachment process on stainless steel surface. *P. aeruginosa* tagged with EGFP were grown for 2 days under flow conditions (flow velocity: 0.5 mm s^{-1}) on stainless steel by using the Stickable Flow Device. The movie begins 15 min after exposure to 0.1% (v/v) Tween 20 under higher flow conditions (flow velocity: 1.5 mm s^{-1}). The 3D structure of the biofilm and stainless surface were observed every 7.5 min for 60 min, from which the panels in Fig. 2E were obtained.

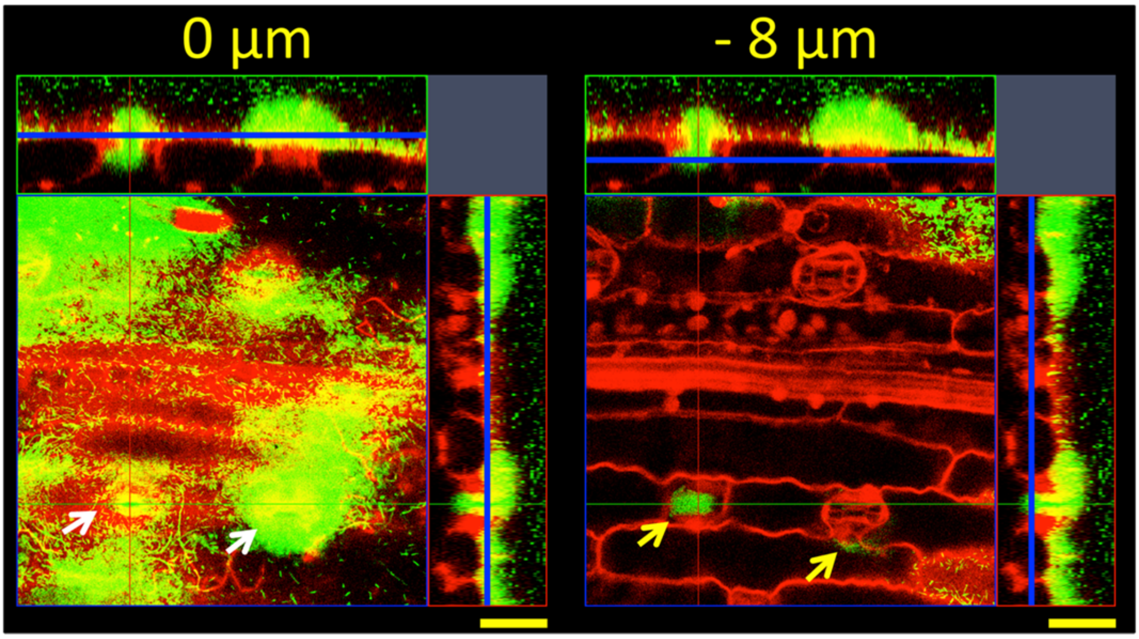

Fig. S7. Cross-sectional images of leaf cell structures and *P. aeruginosa* tagged with EGFP biofilms (green). For detailed observation of leaf cell structures, biofilms and leaf surfaces were stained with propidium iodide (red) (Molecular Probes, Eugene, OR, USA). The horizontal sectioned images (square) and vertical sectioned (rectangle) images were shown. The blue line in the side view images marks the location of the horizontal section. White arrows indicate stomata. Yellow arrows indicate invasion of bacterial cells to apoplastic space located under the stomata. Z-position from the outer leaf surface (top). Scale bar indicates 20 µm. Propidium iodide were illuminated with a HeNe laser (543 nm), and reflected light was collected through a 560-long-pass filter.

References

1. Holloway B.W., V. Krishnapillai, and A.F. Morgan. 1979. Chromosomal genetics of *Pseudomonas*. Microbiol. Rev. **43**:73-102.
2. West S.E.H., H.P. Schweizer, C. Dall, A.K. Sample, and L.J. Runyen-Janecky. 1994. Construction of improved *Escherichia-Pseudomonas* shuttle vectors derived from pUC18/19 and sequence of the region required for their replication in *Pseudomonas aeruginosa*. Gene **148**:81-86.
